# Constructions in Minimalism: A Functional Perspective on Cyclicity

**DOI:** 10.3389/fpsyg.2020.02152

**Published:** 2020-09-04

**Authors:** Andreas Trotzke

**Affiliations:** ^1^Department of Linguistics, University of Konstanz, Konstanz, Germany; ^2^Center for Theoretical Linguistics, Autonomous University of Barcelona, Barcelona, Spain

**Keywords:** construction, cyclicity, derivation, discourse, minimalism, opacity, phases, syntax

## Abstract

This article presents a perspective on syntactic cyclicity in minimalism that is compatible with fundamental ideas in construction–grammar approaches. In particular, I outline the minimalist approach to syntactic structure building and highlight that units of potentially any phrasal size can be atomic items in the syntactic derivation, showing that the opposition between simplex linguistic items (“words”) and more complex ones (“phrases”) in minimalism is in principle as artificial as in many construction–grammar approaches. Based on this perspective on structure building, I focus on the empirical domain of subextraction patterns out of complex subjects, adjuncts, and complements, and I demonstrate that the acceptability patterns in this domain can be explained by a functional approach to syntactic cyclicity: Unacceptable patterns are ruled out not for configurational (and hence syntactic) reasons, but rather they systematically follow from infelicitous interpretations at the syntax–discourse interface. This raises the question of whether syntactic cyclicity is (at least in part) motivated by performance (read: “language-in-use”) constraints, which I consider another area for fruitful interaction between construction–grammar and usage-based accounts on the one hand and minimalism on the other hand.

## Introduction

Generative syntax and construction–grammar approaches share not only their history (Harris, 1993), but also many of their conceptual foundations (see, e.g., Goldberg, 2006, p. 4). I always viewed generative syntax and construction grammar as complementing each other. In this article, I show that “constructions” (read: “indivisible associations between form and meaning,” Fried, 2015, p. 974) are already parts of basic structure building in minimalism. I will illustrate that the set of atomic or “indivisible” items in a derivation not only can consist of words and idiomatic expressions, but also potentially any phrasal unit can become such an atomic item. That is, the opposition between (non-complex, simplex) words and complex phrases is artificial in minimalist structure building too, just like in construction–grammar approaches. To be sure, complex items are generated in generative syntax, whereas they are partially or completely stored in construction grammars. However, “narrow syntax” in minimalism (i.e., the operation “Merge;” Trotzke and Zwart, 2014) in many cases deals with words and complex phrases alike (and relies on “labeling”/a “labeling algorithm” to do that; Chomsky, 2013; Rizzi, 2015). In other words, both words and phrases can be treated as equally atomic for syntactic purposes after they have been merged (cf. also Chomsky’s, 2005 No-Tampering Condition in this context), and I would like to point out in this contribution that this illustrates how the notion of *construction* could be incorporated in the minimalist framework.

The article is structured as follows. The section on *The Numeration and Derivation Layering* first introduces the account of syntactic structure building summarized in Trotzke and Zwart (2014). In the following section on “Syntactic Cyclicity and the Syntax–Discourse Interface,” I focus on subextraction patterns because this empirical domain is one of the key phenomena where members of a separate derivational layer are invisible to syntactic operations in the next layer and can thus count as atomic/indivisible items. I provide a functional account for this chunking operation and argue that derivation layering in subextraction patterns is in many cases determined by discourse rather than by syntactic categories. The section *Conclusion and Outlook* concludes the article and suggests further conceptual overlaps with construction–grammar and usage-based accounts.

## Constructions in Minimalism and Their Functional Motivation

While a lot of generative work has been published on how to best formulate the basic combinatorial operation Merge (see Fukui, 2011; Tanaka et al., 2019 for recent empirical work), there is less research on the question of where the elements to be merged actually come from (or, more accurately, the existing research on Merge and its domains has other ways to frame the question; see section “The Numeration and Derivation Layering”). Because clauses and complex phrases contain words, the simplest suggestion would be that the domain of Merge is the Lexicon. I will demonstrate that the domain of Merge must actually be a set of elements that is much more diverse than just a set of words: The set of elements can also contain phrasal *constructions* in the sense that these items might be internally complex, but are nevertheless dealt with as indivisible atomic items in the course of a derivation. In section “Syntactic Cyclicity and the Syntax–Discourse Interface,” I ask whether “we” can identify a functional motivation of (at least some of) the cases where phrasal units are indivisible items in the derivation, and I will illustrate such a functional account for the empirical domain of subextraction patterns by showing that those patterns can be explained in pragmatic terms rather than in terms of syntactic categories.

### The Numeration and Derivation Layering

**FIGURE 1:**
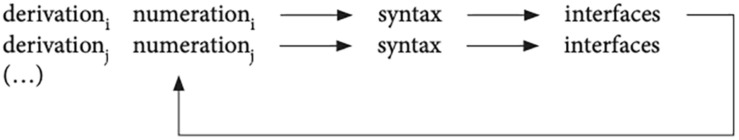
The architecture of derivation layering.

In minimalism, the set of items syntactic derivations draw from is called “numeration” (Chomsky, 1993), to distinguish it from the simplistic concept of a lexicon. One obvious case showing that the domain of Merge can include not only words but also more complex items are idioms such as *kick the bucket*, which refers to an atomic concept (DIE), but nevertheless features regular verb-phrase syntax. There are many ways to deal with idioms in minimalism (see Nediger, 2017 for a recent overview). However, assuming the standard generative model of grammar, where syntax feeds two interface components dealing with sound and meaning, associating the phrase *kick the bucket* with the concept DIE cannot be derived from how the phrase is put together in the syntax. This already indicates that syntax might be connected to the interfaces not only at the end of a syntactic derivation, but also dynamically interacts with them throughout the whole derivation (see Figure 1, from Trotzke, 2015, p. 93). In what follows, I will use the term “derivation layering” for those interactions – a term that has been introduced by Zwart (2009, et seq.).

Crucially, a dynamic system where derivations can be layered and interact with each other via the interfaces is in accordance with minimalist approaches, which assume a cyclic organization of grammar (Nunes and Uriagereka, 2000; Stroik and Putnam, 2013; Trotzke and Zwart, 2014). In particular, in minimalism, “Merge always applies at the simplest possible form: at the root” (Chomsky, 1995, p. 248), and this “Extension Condition” determines that syntax often has to deal with more than one root syntactic object. Let us look at the following derivation of *The man left* (Trotzke and Zwart, 2014, p. 144–146), where we see that the Extension Condition prevents a derivation where *man* in (1d) first merges with *left* because in this case *the* would have to be merged with *man* in a non-cyclic manner, violating the Extension Condition. As a consequence, *the* has to be merged with *man* in a separate derivation layer to form the complex subject [*the man*] (1e)^1^.


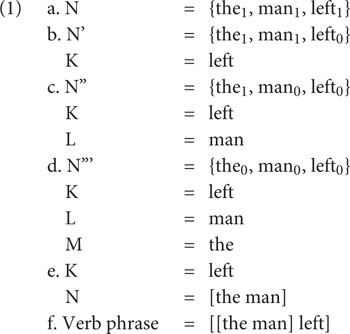


Arguably, the layered derivation architecture shares basic assumptions with alternative frameworks where “combinatorial interface rules” can combine constituents of any size – “whether the constituent C is an utterance, a phrase, or a word” (Jackendoff and Wittenberg, 2014, p. 70); for more discussion, see Trotzke (2015, Chapter 5). In (1), this constituent is the complex subject [*the man*], and the crucial point is that it has to be merged into the derivation as an atom, just like single words (e.g., *left*) enter the derivation. As far as Merge is concerned, there is thus no categorial distinction between words and phrases because complex phrases can serve as atomic “syntactic objects” as well, once they have been derived separately and interpreted at the interfaces. The main idea in standard minimalist derivations like the one in (1) is that Merge is blind to the categorial status and the internal structure of the items it combines and that there is thus no opposition between simplex syntactic objects (also known as “words”) and phrases in syntax proper. Given this perspective, *constructions* are an integral part of minimalist syntax too, as outputs of separate derivation layers – and I hasten to add that we find this core assumption in many more generative approaches, such as nanosyntax (Caha, 2009; Starke, 2010), distributed morphology (Harley and Noyer, 1999; Halle and Marantz, 2004), and related derivational approaches (Marantz, 1997).

The crucial question now is how this derivation layering is motivated on general grounds. Given minimalist methodology (Hornstein et al., 2005), we certainly do not want anything like an “intelligent” spell-out mechanism that would count as a separate module of the grammar. Rather, the numeration, as it is conceptualized in minimalism, is exhaustively determined before the derivation; i.e., it contains all the lexical items and even “subnumerations,” determining opaque domains/“phases” (Chomsky, 2000). There are many questions as to how numerations are put together themselves (cf., e.g., Collins, 1997; Chomsky, 2000). However, this question need not concern us here: In what follows, I will point out how derivations (that only start out from numerations) create domains that are treated as opaque, and that these domains may not have to be defined by formal syntactic means, but follow from more functional (pragmatic and discourse-oriented) factors^2^.

### Syntactic Cyclicity and the Syntax–Discourse Interface

Let us now turn to the key phenomenon of subextraction patterns, which have also been investigated in construction–grammar frameworks (Goldberg, 2013). For reasons of space, I leave it to future research to explore whether the discourse-oriented approach presented here can be extended to related accounts (see Bianchi and Chesi, 2014; Szabolcsi and Lohndal, 2017; Kush et al., 2018, 2019).

The data in (3)–(5) have been used over and over in the generative literature to motivate a syntactic account of subextraction (e.g., Huang, 1982; Uriagereka, 1999). We see that subextraction out of subjects (3b) and adjuncts (4b) is illicit, whereas it is licit in complement cases (5b):


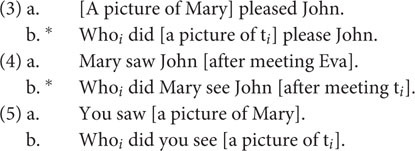


However, many examples indicate that syntactic distinctions (like specifiers vs. complements) cannot be the whole story for explaining subextraction patterns at the clausal level. For instance, Stepanov (2007) has argued that subjects become opaque domains as a result of being moved. Accordingly, when subjects stay *in situ*, extraction out of subjects is allowed:


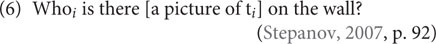


Also, we observe acceptable extractions out of adjuncts (7a) and unacceptable extractions out of complements (8):


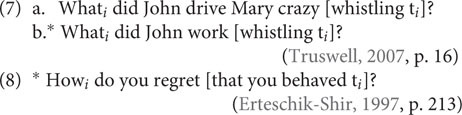


Crucially, we cannot account for (6)–(8) by only referring to configurational criteria and the syntactic status of the extraction domain (i.e., complex left-branch elements such as subjects or adjuncts, or complex complements). Instead, I argue that the subextraction patterns are actually a consequence of discourse constraints.

Let us first turn to extraction out of subjects. I will illustrate my argument based on German data because German is rich in discourse-related syntactic operations (certain scrambling options) that can provide a more fine-grained view on explaining subextraction. The following data are experimentally confirmed by Jurka (2010, 2013): Extraction out of subjects that appear to the right of a German discourse particle such as *denn* (9a) is indeed more acceptable than out of subjects that appear to the left of such a particle (9b); *was-für* split is considered a reliable diagnostic for identifying extraction domains in German:


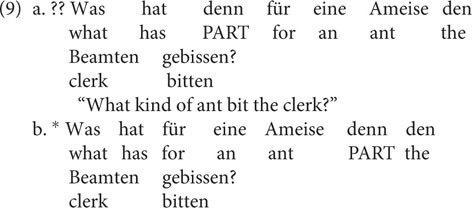


Note that the different placement of the indefinite subject in (9a) vs. (9b) has a discourse effect: As soon as the indefinite subject appears to the left of the particle (9b), it receives a topical interpretation (Bayer and Obenauer, 2011; Bayer and Trotzke, 2015). My point is that the pattern in (9) could be taken to show that it is illicit to extract from a topical constituent. Witness also that Müller (2010) has shown that *was*-*für* splits out of external subjects improve when the object scrambles across the subject (10b). In this example, scrambling the object (*den Fritz*) results in a syntactic configuration that is preferred in cases where the subject (*was für Bücher*) is interpreted as focal; this is due to a complex interplay between syntax, prosody, and pragmatic interpretation (see Struckmeier, 2017 on this point):


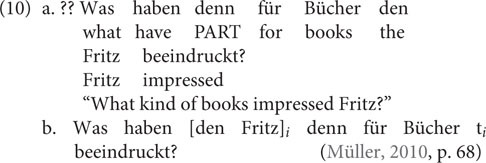


Accordingly, one could also explain subextraction patterns out of subjects along the lines of recent construction–grammar approaches (Ambridge and Goldberg, 2008; Goldberg, 2013): in illicit subject extraction patterns such as the ones listed above, the speaker is treating an element as backgrounded and focal at the same time. This automatically follows if we assume that constituents conveying new information allow extraction the most easily, and elements occurring later in the string (usually the object) are canonically more likely to be interpreted as foci, whereas earlier constituents (usually the subject) are canonically interpreted as topics (see Goldberg, 2006, Ch. 7).

It is now easy to see that such a discourse-oriented approach in terms of backgrounded and focal information could also explain what we observe in the domain of extraction out of complements. Again, observe the following illicit subextraction patterns:


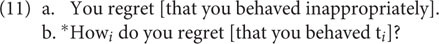


From a discourse-perspective, (11b) is odd because of the semantics of *regret* (and its factivity presupposition); there is a conflict at the syntax–discourse interface between treating an element as at once backgrounded and discourse-prominent. The manner component expressed by *inappropriately* and *how*, respectively, is part of the backgrounded information (the presupposition) and can thus not be highlighted as discourse-prominent in a *wh*-question like (11b). This illustrates that the conflict cannot be explained by syntactic notions such as specifier, adjunct, and complement, but rather the patterns seem to be the result of conflicts that are pragmatic in nature.

Last but not least, let us now see whether a discourse-based explanation can be used for explaining subextraction out of adjuncts as well. In the context of adjunct opacity such as (4) above, it has long been noted that not all adjuncts constitute syntactic islands (see Chomsky, 1982, p. 72):


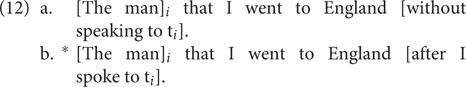


The facts we see in (12) can be explained by the distinction between untensed adjuncts and tensed adjuncts (Cinque, 1990). However, and interestingly, Truswell (2007, et seq.). reported the following patterns of extractability from untensed adjuncts (“Bare Present Participial Adjuncts”):


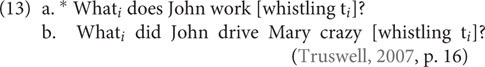


Because an explanation in terms of “tensed vs. untensed adjuncts” will not do the job for those differences, Truswell (2007, p. 150) formulated the “Single Event Condition,” essentially stating that extracting out of an adjunct clause is possible when only a single event is asserted. Accordingly, in both (12b) and (13a), there is a clash at the syntax–discourse interface because the speaker places discourse prominence on a part of the utterance (referred to and highlighted by the pronoun *what*) that is not part of the “macroevent” [e.g., *working* in (13a)], but rather a component of a separate “microevent” (e.g., *whistling*). This microevent is certainly pragmatically backgrounded in the assertion of the macroevent, and so the same discourse conflict as in the cases of subextractions out of subjects and complements arises (see above).

To sum up, in the context of subject, adjunct, and complement opacity, the chunking of the derivation into opaque domains is determined by properties of the syntax–discourse interface: A single derivation layer cannot contain two syntactic objects whose interpretations clash at the syntax–discourse interface (pointing to something as discourse prominent and backgrounded at the same time). In contrast to common minimalist approaches to syntactic cyclicity (e.g., phase theory), I thus suggest that the opacity of a syntactic domain is not necessarily determined by that domain’s *syntactic category*, but rather in many cases the result of the *discourse status* of that domain.

## Conclusion and Outlook

The section on “Constructions in Minimalism and Their Functional Motivation” has indicated that the opposition between words and phrases in minimalism is artificial in the sense that elements of any size can serve as the building blocks of Merge. This is an assumption of current generative models of grammar. Crucially, this concept opens a path of defining *constructions* in minimalism: They are outputs of separate derivation layers. Moreover, the perspective articulated here has suggested that certain domains of syntactic cyclicity should not be defined in syntactic terms (e.g., in terms of categorial status). Rather, I have illustrated that some (or perhaps many) of those domains can actually be characterized in functional terms such as their status in a discourse. In other words, the impact of how language is used in a context on syntax might not only be seen in marked word order, dislocations, etc. Rather, it also affects the cyclic organization of grammar itself and the domains Merge can operate on.

Last but not least, I would like to highlight in this context that not only “language-in-use” factors such as discourse and pragmatics seem to play a crucial role in minimalism, but also processing-based considerations. Specifically, minimalism often refers to derivations as “actual computations,” and the notion of a “phase” basically (re)introduces the concept that derivations proceed in incremental chunks – and there are, in fact, some recent approaches trying to reconcile processing considerations with phase-based derivations (e.g., Chesi, 2015; Chesi and Canal, 2019). At a more conceptual level, Trotzke et al. (2013) have discussed cases where the nature of syntactic constraints suggests a direct link between grammar and performance systems (like memory constraints). Without having to claim that all of grammar is “usage-based,” minimalists could therefore take seriously the role of the “performance interface,” which might dovetail nicely with minimalist third-factor explanations.

## Data Availability Statement

All datasets presented in this study are included in the article/supplementary material.

## Author Contributions

AT confirms being the sole contributor of this article, and he has approved it for publication.

## Conflict of Interest

The authors declare that the research was conducted in the absence of any commercial or financial relationships that could be construed as a potential conflict of interest.

## Footnotes

1 N is the “numeration” with which a derivation starts. The index number says how often an item occurs in the numeration; the number is zero once the item has been merged; K, L, and M refer to projected or unprojected lexical items (Chomsky, 1993). Note that postulating a numeration is not currently standard in all generative approaches. I will nevertheless use the notion of a numeration here in order to link the presentation to my previous work on this topic (cited above).

2 Further research may show that (sub-)numerations are ultimately responsible for the specific structure so created, but this question is not addressed in this article.
